# Targeting AKT via SC79 for Photoreceptor Preservation in Retinitis Pigmentosa Mouse Models

**DOI:** 10.3390/biomedicines14010195

**Published:** 2026-01-15

**Authors:** Alicia A. Brunet, Kate Gilbert, Annie L. Miller, Rebekah E. James, Xin Ru Lim, Alan R. Harvey, Livia S. Carvalho

**Affiliations:** 1 Lions Eye Institute Ltd., 2 Verdun St, Nedlands, WA 6009, Australia; 2 Centre for Ophthalmology and Visual Sciences, The University of Western Australia, 35 Stirling Hwy, Crawley, WA 6009, Australia; 3 Department of Optometry and Vision Sciences, University of Melbourne, Parkville, VIC 3052, Australia; 4 School of Biomedical Sciences, The University of Western Australia, 35 Stirling Hwy, Crawley, WA 6009, Australia; 5 School of Human Sciences, The University of Western Australia, 35 Stirling Hwy, Crawley, WA 6009, Australia; 6 Perron Institute for Neurological and Translational Science, 8 Verdun St, Nedlands, WA 6009, Australia

**Keywords:** retinitis pigmentosa, photoreceptor degeneration, AKT signalling, neuroprotection, rod photoreceptors, cone photoreceptors, rd1 mouse, *Rho* P23H mouse

## Abstract

**Background/Objectives**: Retinitis pigmentosa is a degenerative retinal disease and a major cause of inherited blindness globally. The pro-survival kinase AKT is downregulated in degenerating photoreceptors in retinitis pigmentosa, and its activation has shown neuroprotective effects in retinitis pigmentosa and other neurodegenerative disorders. In this study, we evaluated the therapeutic potential of SC79, a pharmaceutical AKT activator, in two mouse models of retinitis pigmentosa, *rd1*.GFP and *Rho*P23H.GFP. **Methods**: SC79 was administered intravitreally at postnatal day 12 (P12) and analysis was conducted at P16. **Results**: SC79 at 10 µM was well tolerated in wildtype mice, with no reduction in retinal function or thickness. In *rd1*.GFP mice, SC79 partially preserved peripheral outer nuclear layer (ONL) thickness, improved rod photoreceptor-driven optomotor contrast sensitivity responses, and improved cone photoreceptor morphology. Immunohistochemistry of retinal sections indicated AKT-related protein expression changes in both sham and SC79-treated *rd1*.GFP retinas, with sham injections leading to decreases in this pathway and SC79 injections restoring this back to uninjected protein levels or higher, indicating the damage from intravitreal injections can induce AKT-related protein expression changes. In *Rho*P23H.GFP mice, changes to the visual response from the therapeutic effects of SC79 were not detectable. An increased dosage of SC79 at 100 µM was evaluated in wildtype mice and showed no major toxic effects, although it did not confer neuroprotective benefits in either disease model. **Conclusions**: These results demonstrate the potential therapeutic effect of AKT pathway modulation for preserving photoreceptors in recessive retinitis pigmentosa, with further optimisation of treatment delivery required.

## 1. Introduction

Retinitis pigmentosa is a rod-cone dystrophy and one of the leading causes of inherited retinal disease (IRD) [1]. IRDs affect around 1 in 4000 people worldwide [1], with over four hundred disease susceptibility genes identified [2]. Retinitis pigmentosa is considered a progressive disease in which patients initially exhibit symptoms of night blindness as a result of mutations causing malfunction and degeneration of rod photoreceptor cells [1]. Following loss of the rods, there is a progressive secondary degeneration of cone photoreceptors that are responsible for daylight vision [3]. Worsening of vision begins in the periphery, advancing centrally until vision may be completely lost.

AKT, also known as protein kinase B (PKB), plays an important role in various cellular processes such as metabolism, proliferation, and neuronal survival [4]. In retinitis pigmentosa, AKT has been shown to be deactivated during major photoreceptor degeneration in the *rd1* mouse model of recessive retinitis pigmentosa [5]. The role of AKT in neuronal survival are exemplified in other retinal degenerations, such as diabetic retinopathy, whereby AKT activity is substantially decreased in mice models of diabetic retinopathy [6]. In retinal induced-damage models, its activity was shown to increase in all layers of the retina after optic nerve clamping-induced retinal damage [7]. Inhibition of AKT was shown to further exacerbate retinal ganglion cell death following optic nerve clamping, suggesting that AKT has a neuroprotective effect in retinal degeneration. Activation of AKT using SC79, an AKT agonist, has been shown to protect dopaminergic neurons from oxidative stress [8] and retinal pigment epithelium cells from ultraviolet radiation [9].

In the *rd10* mouse model of recessive retinitis pigmentosa, supplementation of AKT through gene therapy increased visual response and attenuated photoreceptor degeneration [10]. However, translating this therapy clinically would be costly, as gene therapy production is expensive and the ocular procedure would require highly precise subretinal injections of the drug [11]. The cost of Luxturna, the first and only FDA-approved gene therapy that targets one IRD gene (*RPE65*) [12], is approximately $850,000 USD [13]. Thus, AKT activation through pharmaceutical drugs may be a potential cost-effective alternative neuroprotective therapy for retinitis pigmentosa.

Here, we report on the therapeutic potential of targeting AKT via SC79 and its neuroprotective potential to protect degenerating photoreceptors in autosomal recessive and autosomal dominant retinitis pigmentosa mouse models, thereby assessing whether this approach is an effective gene-agnostic therapy across different inheritance patterns. After treatment with SC79 in *rd1*.GFP mice, we observed a partial preservation via improved optomotor response and cone morphology. In contrast, therapeutic benefits were not detectable in the dominant *Rho*P23H.GFP mouse model of retinitis pigmentosa. Thus, the benefits of SC79 were modest, and pharmaceutical targeting of AKT needs to be optimised for improved therapeutic outcomes. The results from this study provide a foundation for further exploration of the therapeutic potential of targeting AKT for photoreceptor neuroprotection in retinitis pigmentosa.

## 2. Materials and Methods

### 2.1. 661W Cell Culture and MTT Assay

Murine cone-like 661W cells were kindly provided by Prof. Muayyad Al-Ubaidi (University of Houston). Cells were routinely cultured in Dulbecco’s Modified Eagle’s Medium (DMEM, Gibco, 11885092, Thermo Fisher Scientific, Waltham, MA, USA) containing 10% foetal bovine serum (FCS, FBS-AU-015, FisherBiotec, Wembley, Western Australia, Australia) and 1% penicillin-streptomycin (Thermo Fisher Scientific, Waltham, MA, USA, 15140122) in T75 flasks, and maintained at 37 °C in a humidified atmosphere containing 5% CO_2_. For drug toxicity testing of SC79, cells were seeded at 5 × 10^3^ into 96-well plates. The outside surrounding wells of each plate were filled with water to prevent evaporation. After plating, cells were left to settle for 24 h before the addition of SC79 at various concentrations.

Cell viability was tested using an MTT assay (11465007001, Sigma-Aldrich, Burlington, MA, USA). Then, 24 h after the addition of drugs, media was aspirated to leave a remaining 100 µL in wells and 10 µL of MTT was added. Cells were incubated in MTT for 3 h at 37 °C. The remaining media was removed and 100 µL of dimethyl sulfoxide (DMSO) was added to dissolve the formazan crystals. Absorbance was measured at 570 nm using the Epoch Microplate Spectrophotometer (BioTek, Winooski, VT, USA) and results are presented as a percentage of survival compared to control.

### 2.2. Animals

All mouse lines were bred on a C57BL/6J background and were bred and housed at the Harry Perkins Institute of Medical Research bioresources facility. The animals were housed in a 12/12 h day/night cycle and received ad libitum access to food and water. Mouse experiments were approved by the Harry Perkins animal ethics committee and the University of Western Australia ethics committee (AE301). The Chrnb4.GFP reporter line (STOCK Tg(Chrnb4-EGFP)CL200Gsat/Mmnc, RRID:MMRRC_000259-UNC) was obtained from the Mutant Mouse Resource and Research Centres (MMRRC) at the University of North Carolina at Chapel Hill, an NIH-funded strain repository, and was donated to the MMRRC by Nathaniel Heintz, Ph.D., the Rockefeller University, GENSAT. Chrnb4.GFP mice expressing green fluorescent protein (GFP) in cones [14] was used as the wildtype control. The retinal degeneration 1 (*rd1*) mouse model of retinitis pigmentosa was isolated from the Chrnb4.GFP line as part of a previous C3H/HeJ background. It was confirmed via genotyping to carry the standard *Pde6b* mutations found in C3H/HeJ: the murine leukaemia virus (Xmv-28) insertion in reverse orientation in intron 1 and a nonsense mutation (C-to-A transversion) in codon 347. The *Rho* P23H mouse of autosomal dominant retinitis pigmentosa was purchased from the Jackson Laboratory (#017628) [15] and used in its heterozygous form for the mutation. Using a similar method as described previously [16], the retinitis pigmentosa mouse models were crossbred with wildtype to generate GFP^+^ cones in the retinitis pigmentosa mouse lines (referred to as *rd1*.GFP and *Rho*P23H*.GFP* herein).

### 2.3. Intravitreal Injections

Animals were treated at postnatal day 12 (P12), and further experiments were conducted four days post-injection at P16. Mice received anaesthesia via intraperitoneal injection of Ketamine (40 mg/kg; Ceva Animal Health Pty Ltd., Amersham, UK) and Xylazil-100 (5 mg/kg; Troy Laboratories, Glendenning, NSW, Australia). Pupils were dilated using 1% tropicamide drops. The eye was proptosed and a moisturising eye gel (GenTeal, Alcon, Geneva, Switzerland) was added. A pilot hole was created using a 29-gauge bevelled needle at the temporal pars plana and a 35-gauge blunt needle connected to a syringe was used to deliver the drugs. Assuming a vitreous volume of 4 µL in P12 pups, an intravitreal injection of 0.4 µL was administered, with SC79 (SML0749, Sigma-Aldrich, Burlington, MA, USA) in one eye and a sham injection in the contralateral eye. SC79 was dissolved in 100% DMSO before being diluted to the desired concentration in saline on the day of injection. SC79 at 10 µM dose was dissolved at 20 mg/mL in DMSO and diluted to a final concentration of 0.2% DMSO in solution. SC79 at 100 µM was dissolved at 100 mg/mL in DMSO and diluted to a final concentration of 0.365%. Sham injections consisted of saline and the equivalent amount of DMSO. Reversal of anaesthesia was performed through a subcutaneous injection of Ilium Atipamezole (1 mg/kg; Troy Laboratories, Glendenning, NSW, Australia). Mice were left on a 37 °C heat mat until fully recovered from the anaesthesia.

### 2.4. Electroretinograms (ERGs)

To assess retinal function, electroretinograms (ERGs) were recorded for scotopic (rod-mediated vision) and photopic (cone-mediated vision) responses. Mice were dark-adapted overnight and handled under dim red light for the scotopic paradigm. Mice were anaesthetized through intraperitoneal injection using 80 mg/kg ketamine (Ceva Animal Health Pty Ltd., Amersham, UK) and 10 mg/kg Xylazil-100 (Troy Laboratories, Glendenning, NSW, Australia), before adding 1% tropicamide drops (Alcon, Geneva, Switzerland) to the eye to dilate the pupil. Then, 2% hypromellose (HUB Pharmaceuticals LLC, Scottsdale, AZ, USA) was applied to moisturise the eye and act as a contact fluid. Mice were placed on the Celeris ERG heated platform (Diagnosys, Lowell, MA, USA) and electrodes were positioned in front of the eye, being careful to avoid contact with the cornea to reduce the occurrence of corneal scarring. Readings were measured using 1 millisecond flashes repeated four times and spaced 10 s apart. The different light intensities measured were 0.01, 0.1, 1, 3, 10, and 25 cd.s.m^2^, with 60 s of recovery between different flash intensities. After scotopic readings were completed, mice were light adapted for 10 min at 40 cd.s.m^2^ before commencing photopic readings. Photopic readings were measured at 0.1, 1, 3, 5, 10, and 25 cd.s.m^2^ with a background light intensity of 30 cd.s.m^2^. Data were analysed for amplitudes of a-wave (response of photoreceptors) and b-wave (response of inner retinal cells) responses using the Espion V6 software (Diagnosys, Lowell, MA, USA) and Microsoft Excel (Microsoft 365 MSO, Version 2511 Build 16.0.19426.20186).

### 2.5. Optomotor Response

Optomotor responses were assessed in mice using the automated OptoDrum (Striatech, Tubingen, Germany), a box containing four LCD monitors that simulate a rotating cylinder of alternating white and black square-wave gratings. Mice were individually placed on a raised platform at the centre of the box, with a camera directly above to track temporal-nasal head movements via the software. Mice were dark-adapted overnight before scotopic recordings, followed by light adaptation for 2 h before conducting photopic recordings. Scotopic conditions assessed rod-mediated responses by contrast sensitivity under 2 mlux, whereby the contrast between black and white stripes ranged from 100% to 0%. Photopic conditions assessed cone-mediated responses by visual acuity under 70 lux, whereby the spatial frequency between black and white stripes ranged between 0.556 and 0.0556 cycles/degree. Mice were tested in each condition until there was an absence of a head movement reflex, determined by the software if the head movement score no longer exceeded the chance level of a stimulus-independent head movement. The velocity of the virtual cylinder rotation was kept constant at 12°/s.

### 2.6. Immunohistochemistry

Immunohistochemical staining was conducted on frozen retinal sections. To process eyes for sectioning, eyes were enucleated and fixed in 4% *v*/*v* paraformaldehyde at room temperature for 1 h, with the cornea being removed at 30 min incubation during this time. After fixation, eyes were then cryoprotected overnight in 20% *w*/*v* sucrose solution at 4 °C. The lens was removed, and the remaining eyecup was embedded in optimal cutting temperature (4583, Tissue-Tek, Torrance, CA, USA) compound and frozen with isopentane (320404, Sigma-Aldrich, Burlington, MA, USA) submerged in liquid nitrogen (N_2_). Eyecups were sectioned on a cryostat (CM3050S, Leica, Wetzlar, Germany) at a 14 µm thickness, and sections placed on frosted microscope slides (SCF90W-PC, Hurst Scientific, Forrestdale, WA, Australia). Sections were blocked for 1 h in blocking buffer (1% *w*/*v* bovine serum albumin [BSA; Bovogen Biologicals, Keilor East, VIC, Australia, BSAS0.1], 0.5% *v*/*v* Trion-X-100 [1552–500 mL, LabChem, Zelienople, PA, USA], and 5% *v*/*v* normal goat serum [NGS; G9023, Sigma-Aldrich, Burlington, MA, USA], in 1X PBS) at room temperature. Primary antibodies diluted in blocking buffer were added to the sections and were incubated overnight at 4 °C (AKT 1:250, Cell Signalling Technologies, Denver, MA, USA, 9272S; p-AKT Ser473 1:250 Cell Signalling Technologies, 4060T; p-mTOR Ser2448 1:500, Abcam, Cambridge, UK, ab109268; and GFAP 1:500, Dako, Glostrup, Denmark, Z0334). Following overnight incubation, sections were washed three times with 1X PBS before addition of the secondary antibody (AlexaFluor-568 1:500, Abcam, Cambridge, UK, ab175471) diluted in blocking buffer and were then left to incubate for 2 h at room temperature. Sections were counterstained with 4′,6-diamidino-2-phenylindole (DAPI) for 5 min at room temperature and visualised on a Nikon (Shinagawa, Japan) AX confocal microscope.

### 2.7. Fluorescence Intensity Quantification

Z-stack images were acquired at 2 µm step intervals using a 20X dry objective for GFAP staining and 40X dry objective for AKT, p-AKT, and p-mTOR staining. Three adjacent sections close to the optic nerve per sample at superior and inferior retinal portions were imaged, resulting in six images per sample that were averaged for the final results. Individual sections were excluded from final analysis if there was too much damage from tissue processing. Fluorescence intensity was measured from the internal limiting membrane to the external limiting membrane or photoreceptor outer segment as the region of interest depending on the expression pattern using the ImageJ software v1.53c.

### 2.8. Retinal Thickness Quantification

Samples for quantification were blinded prior to imaging to eliminate bias. Quantitative images were originally captured using a Nikon Eclipse N*i* fluorescent microscope on a 20X dry objective. Each biological replicate had two adjacent retinal sections imaged which were close to the optic nerve. Each section had the following four positions imaged relative to the optic nerve: +80° (superior peripheral, SP), +10° (superior central, SC), −10° (inferior central, IC), and −80° (inferior peripheral, IP). The length of the ONL and INL were manually measured from each region by averaging four equidistant measurements within each image using the NIS-elements AR 5.42.01 software. Representative images were taken using a Nikon AX confocal microscope.

### 2.9. Cone Photoreceptor Quantifications

Cone numbers were quantified using flow cytometry through identification of the GFP^+^ population. Fresh retinas from each mouse line were dissected out of the eye and dissociated with a papain digestion described previously [16]. Total cell numbers were analysed on a Z Series Coulter Counter (Beckman Coulter, Brea, CA, USA). Flow cytometry was performed on the BD FACSMelody™ Cell Sorter (Franklin Lakes, NJ, USA) to quantify the percentage of GFP^+^ cells in each sample, representing percentage of cone photoreceptors.

### 2.10. Statistical Analysis

Data were analysed using the GraphPad Prism software v9.5.1. A one-way ANOVA and Dunnett’s multiple comparisons test were used to analyse 661W cell data. Statistical tests of all mouse experiments included two-way ANOVA and a Sidak’s multiple comparisons test when comparing across two groups, or Tukey’s multiple comparisons test when comparing across three or more treatment groups. Data were assumed to be statistically significant if *p* < 0.05.

## 3. Results

### 3.1. SC79 Treatment in Healthy Conditions

**Figure 1:**
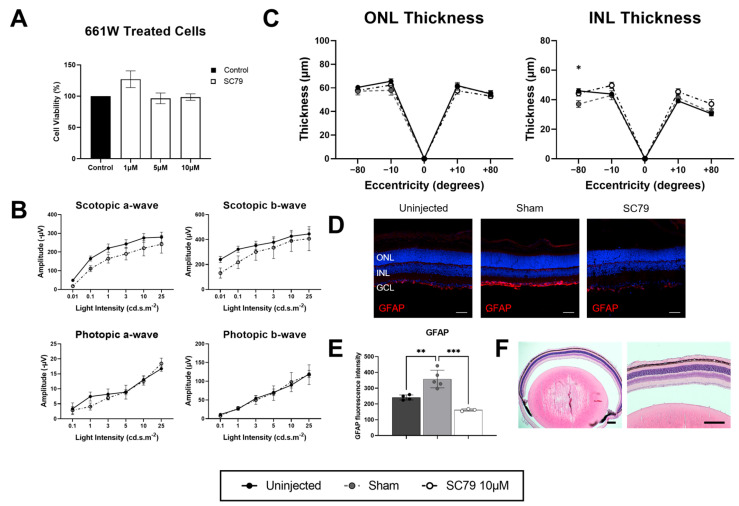
Safety testing of SC79 in healthy conditions. (**A**) 661W cell viability after SC79 treatment at different concentrations (1 µM, 5 µM, and 10 µM) compared to untreated controls using MTT assay. Numbers are mean ± SEM. *n* = 3 experimental replicates. (**B**–**F**) Wildtype mice treated at P12 with SC79 10 µM, with eyes analysed at P16. (**B**) ERG responses in SC79 10 µM treated wildtype mice compared to uninjected controls. Numbers are mean ± SEM. Uninjected *n* = 4; SC79 10 µM *n* = 3. (**C**) Retinal ONL and INL thickness in uninjected, sham-injected, and SC79 10 µM-injected wildtype mice. Numbers are mean ± SEM. Uninjected *n* = 5; sham *n* = 5; SC79 10 µM *n* = 6. Uninjected compared to sham-injected wildtype mice; * *p* < 0.05. ONL: outer nuclear layer; INL: inner nuclear layer. (**D**) GFAP staining (red) showing glial reactivity in uninjected, sham-injected, and SC79 10 µM-injected wildtype mice. Representative images were taken from the medial retina. Scale bar = 50 µm. (**E**) Analyses of relative GFAP fluorescence intensity in retinal sections from each group are shown. Fluorescence intensity was quantified by averaging three retinal sections per eye, with superior and inferior regions analysed for each section and averaged for the final value. Numbers are mean ± SEM. Uninjected *n* = 4; sham *n* = 5; SC79 10 µM *n* = 3. ** *p* < 0.01, and *** *p* < 0.001. (**F**) H&E stained paraffin section of SC79 10 µM-treated wildtype mouse showing no infiltration of immune cells in the vitreous humour. Scale bar = 200 µm.

Before commencing in vivo experiments, we tested the potential toxicity of SC79 in the cone photoreceptor-like 661W cells grown in regular culture conditions (Figure 1A). Concentrations tested (1 µM, 5 µM, and 10 µM) were chosen based on a previous study which treated primary neuronal cultures with SC79 at 10 µM [8]. After 24 h of treatment, MTT assays showed no significant change in cell viability compared to untreated controls. In vivo safety testing was assessed by intravitreal injections of SC79 at 10 µM in wildtype mice at P12, with eyes collected for analysis four days post-treatment at P16. Electroretinogram (ERG) responses revealed no significant changes after treatment in either scotopic or photopic conditions, though there was a potential trend for decreased scotopic responses after SC79 treatment (Figure 1B). Additionally, retinal thickness was quantified via histology, where we found no major decreases to outer nuclear layer (ONL) or inner nuclear layer (INL) thickness after treatment (Figure 1C). The impact of the treatment on retinal health was further assessed through Müller glia and astrocyte reactivity, with glial fibrillary acidic protein (GFAP) staining and fluorescence quantification showing a significant increase in sham-injected mice (Figure 1D,E), suggesting potential negative effects of the sham injection on glial response. However, SC79-treated mice had comparable levels of GFAP expression to uninjected controls. Paraffin sections stained with H&E showed no obvious infiltration of immune cells into the vitreous humour in SC79 treated wildtype mice (Figure 1F), thus showing SC79 to have a high safety profile at the 10 µM dose.

### 3.2. SC79 Treatment in Retinitis Pigmentosa Mice

**Figure 2:**
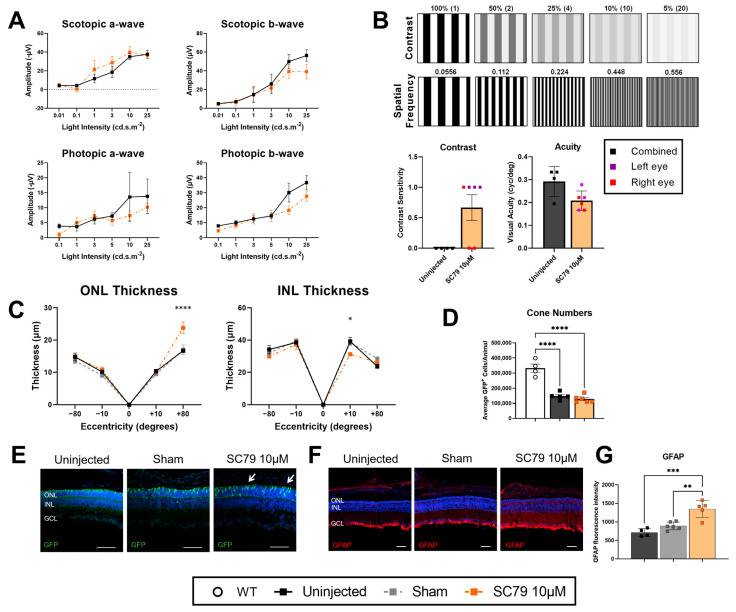
The effect of SC79 treatment on visual response, photoreceptor viability, and cone morphology in *rd1*.GFP mice. (**A**) ERG responses in SC79 10 µM-treated *rd1*.GFP mice compared to uninjected controls. A black dotted line is shown at y = 0 when the lower bound of the error bars extends below zero. Uninjected *n* = 4; SC79 10 µM *n* = 4. (**B**) Optomotor responses for contrast sensitivity under scotopic conditions and visual acuity under photopic conditions (cycles per degree, cyc/deg) in *rd1*.GFP mice after treatment, compared to uninjected controls. SC79 10 µM +-treated left and right eye were analysed independently to control for injection bias (*n* = 6 eyes). Uninjected controls used the combined response of left and right eyes (*n* = 4 animals). (**C**) Retinal ONL and INL thickness measurements in uninjected, sham-injected, and SC79 10 µM-injected *rd1*.GFP mice. Numbers are mean ± SEM. Uninjected *n* = 5; sham *n* = 7; SC79 10 µM *n* = 4. * *p* < 0.05, **** *p* < 0.0001. ONL: outer nuclear layer; INL: inner nuclear layer. (**D**) Cone number quantifications using flow cytometry of GFP^+^ cells in age-matched wildtype mice, and uninjected and SC79 10 µM injected *rd1*.GFP mice. Numbers are mean ± SEM. WT *n* = 4; Uninjected *n* = 5; SC79 10 µM *n* = 6. **** *p* < 0.0001. (**E**) Representative images of cone morphology in SP retina *rd1.*GFP mice after SC79 10 µM treatment. White arrows indicate partial preservation of cone inner segments. Scale bar = 50 µm. (**F**) GFAP staining (red) showing glial reactivity in uninjected, sham-injected, and SC79 10 µM-injected *rd1*.GFP mice. Representative images were taken from the medial retina. Scale bar = 50 µm. (**G**) Analysis of relative GFAP fluorescence intensity in retinal sections from each group are shown. Fluorescence intensity was quantified by averaging three retinal sections per eye, with superior and inferior regions analysed for each section and averaged for the final value. Numbers are mean ± SEM. Uninjected *n* = 4; sham *n* = 6; SC79 10 µM *n* = 5. ** *p* < 0.01, *** *p* < 0.001.

As minimal negative effects were observed in wildtype mice after SC79 treatment, SC79 was administered to the autosomal recessive retinitis pigmentosa *rd1*.GFP mouse at P12 at the same concentration (10 μM) to assess the therapeutic effects of the drug (Figure 2). ERG responses four days post-treatment showed no significant difference between SC79-treated and uninjected controls (Figure 2A). Despite limited changes to visual response as assessed by ERG measurements, optomotor response data showed that treated *rd1*.GFP mice regained some level of scotopic contrast sensitivity (Figure 2B). Left and right eyes were measured separately in treatment groups to avoid bias that could be caused by injections, whilst uninjected results used the average of both eyes with the assumption that there was an absence of bias between left and right eye responses. Contrast sensitivity under scotopic conditions were unrecordable in uninjected *rd1.*GFP mice at P16. However, some *rd1*.GFP mice recovered measurable contrast sensitivity after treatment with SC79. Visual acuity (cyc/degree) in photopic conditions was unchanged across uninjected and treatment groups. Retinal thickness analysis showed that the superior peripheral (SP) region of the ONL was significantly increased in SC79-treated *rd1*.GFP mice compared to uninjected controls, though not significantly increased when compared to sham-treated mice (Figure 2C). There was also a significant decrease in the superior central (SC) region in the INL in treated *rd1*.GFP mice. Visual representation of retinal thickness for each group via immunohistochemistry is provided in Supplementary Figure S1. To investigate the effects of SC79 specifically on cones, flow cytometry was conducted at P16 between wildtype, *rd1.*GFP uninjected, and *rd1*.GFP SC79-treated mice, and showed no improvement in cone numbers after treatment (Figure 2D). Interestingly, while cone numbers did not appear to increase, it was noted that cone morphology was improved after SC79 treatment, with this being most apparent in the superior peripheral (SP) retina where inner segments were moderately rescued from degeneration (indicated by white arrows). Previous studies have shown that *rd1* mice undergo an increased glial reactivity as part of the degenerative process [17]. SC79-treated *rd1*.GFP mice had a significantly higher expression of GFAP compared to uninjected and sham-injected controls (Figure 2F,G).

**Figure 3:**
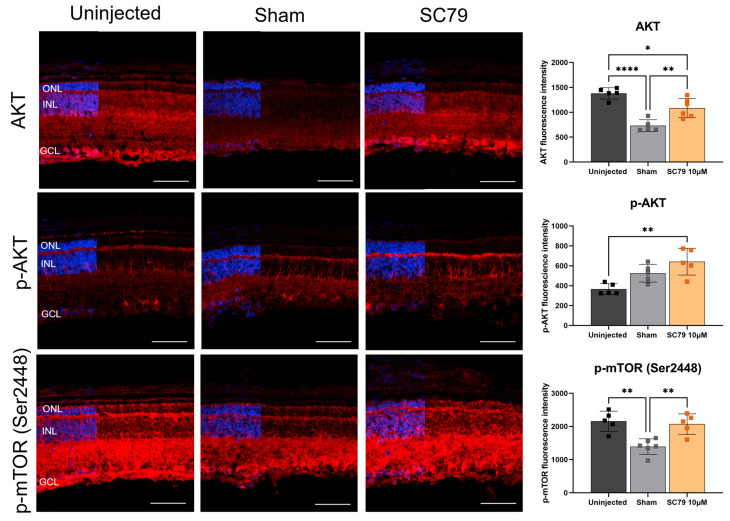
The effect of SC79 treatment on AKT-related protein expression in *rd1*.GFP mice. Retinal sections were stained either with AKT (**top**), p-AKT (**middle**), or p-mTOR (Ser2448) (**bottom**) and visualised under a red channel. All representative images were taken from the superior peripheral retina. ONL: outer nuclear layer; INL: inner nuclear layer; and GCL: ganglion cell layer. Scale bar = 50 µm. Analysis of relative fluorescence intensity in retinal sections from each group are shown. Fluorescence intensity was quantified by averaging three retinal sections per eye, with superior and inferior regions analysed for each section and averaged for the final value. Numbers are mean ± SEM. Uninjected *n* = 5; sham *n* = 5 for AKT and p-AKT, *n* = 6 for p-mTOR (Ser2448); SC79 10 µM *n* = 5. * *p* < 0.05, ** *p* < 0.01, and **** *p* < 0.0001.

To assess changes to AKT protein expression after SC79 treatment, retinal sections were stained for AKT (Figure 3). AKT expression was identified throughout the retina of uninjected *rd1*.GFP mice, most notably in the ganglion cell layer. After sham treatment, AKT expression was significantly lower. Interestingly, SC79-treated mice had higher AKT expression than sham-treated mice, but slightly lower expression than uninjected mice. The activated form of AKT, which is phosphorylated at Ser473 (p-AKT), was significantly increased in SC79-treated groups compared to uninjected controls, and trended higher in expression than sham-injected mice, though this was not found to be significant. It is important to note that a downstream effector of p-AKT is the mammalian target of rapamycin (mTOR) which becomes phosphorylated at Ser2448 (p-mTOR), resulting in the activation of mTOR [18]. The expression of p-mTOR (Ser2448) followed a similar pattern to AKT expression, in that there was widespread expression throughout uninjected retinas and decreased expression in sham. Expression of p-mTOR (Ser2448) was comparable between uninjected and SC79-injected groups. These findings verified the mode of action of SC79 on the AKT pathway, showing protein expression remained altered even four days post-injection.

**Figure 4:**
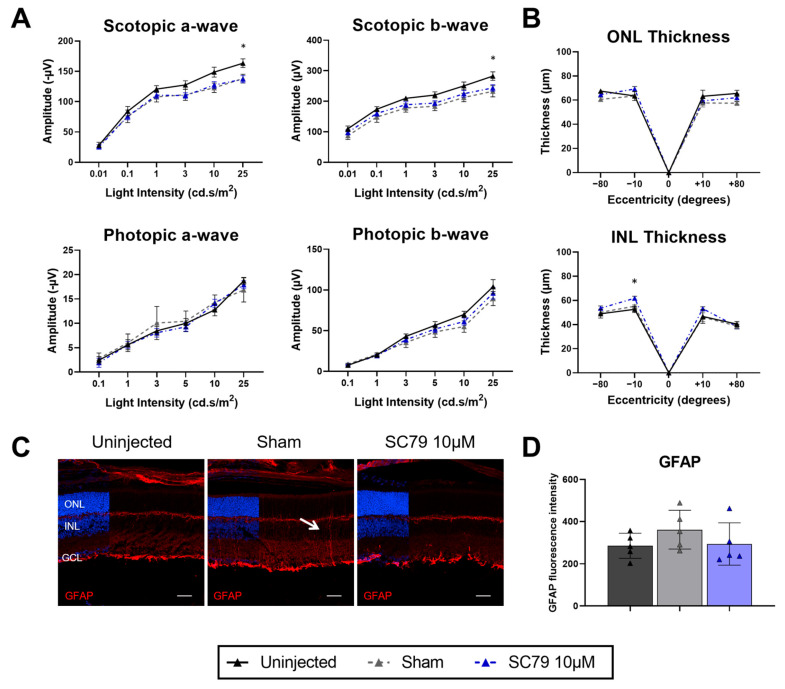
SC79 treatment in *Rho*P23H.GFP mice. (**A**) ERG responses in SC79 10 µM-treated *Rho*P23H.GFP mice compared to uninjected controls. Uninjected *n* = 8; SC79 10 µM *n* = 9. Numbers are mean ± SEM. * *p* < 0.05. (**B**) Retinal ONL and INL thickness measurements in uninjected, sham-injected, and SC79 10 µM-injected *Rho*P23H.GFP mice. Numbers are mean ± SEM. Uninjected *n* = 6; Sham *n* = 5; SC79 10 µM *n* = 9. * *p* < 0.05. ONL: outer nuclear layer; INL: inner nuclear layer. (**C**) GFAP staining (red) showing glial reactivity in uninjected, sham-injected, and SC79 10 µM-injected *Rho*P23H.GFP mice. Representative images were taken from the medial retina. White arrow indicates extension of GFAP positive Müller glia processes. Scale bar = 50 µm. (**D**) Analysis of relative GFAP fluorescence intensity in retinal sections from each group are shown. Fluorescence intensity was quantified by averaging three retinal sections per eye, with superior and inferior regions analysed for each section and averaged for the final value. Numbers are mean ± SEM. Uninjected *n* = 5; sham *n* = 5; SC79 10 µM *n* = 5.

As we found some therapeutic effects in *rd1*.GFP mice, an autosomal recessive retinitis pigmentosa model, we investigated the effects of SC79 in the *Rho*P23H.GFP autosomal dominant mouse model to assess whether benefits extend to other inheritance forms of the disease (Figure 4). In contrast to *rd1*.GFP mice, *Rho*P23H.GFP mice used within this study were heterozygous for the disease mutation, with major rod degeneration only occurring after 1 month of age [15]. Despite the relative preservation of rods at early timepoints, ERG responses are reported to be significantly reduced in *Rho*P23H.GFP from early ages [19]. Given the slower progression of rod degeneration in this model, any therapeutic effects of a single intravitreal injection may not produce detectable changes in retinal thickness before the drug is cleared from the eye. To determine whether decreased visual function independent from significant rod degeneration could be restored, we performed injections at P12 in *Rho*P23H.GFP to reflect the *rd1*.GFP treatment groups injection age. Four days post-treatment, there were significant decreases to scotopic ERG responses at 25 cd.s/m^2^ between SC79 treated and uninjected *Rho*P23H.GFP controls, though photopic responses followed uninjected levels (Figure 4A). Retinal thickness was mostly unaffected after treatment with sham or SC79, with only the inferior central retina INL exhibiting significant increase (Figure 4B). It is important to note that previous studies have indicated the optomotor responses [20] and cone numbers [21] in *Rho^P23H/WT^* mice are unaltered compared to wildtype at early ages, so these measures were not assessed. GFAP positive staining of Müller glial processes across retinal layers was observed in sham-injected *Rho*P23H.GFP, but not seen in uninjected and SC79-treated groups; however, GFAP expression was not significantly increased in sham-injected mice (Figure 4C,D).

### 3.3. Effects of Increased SC79 Dose in Healthy Mice

**Figure 5:**
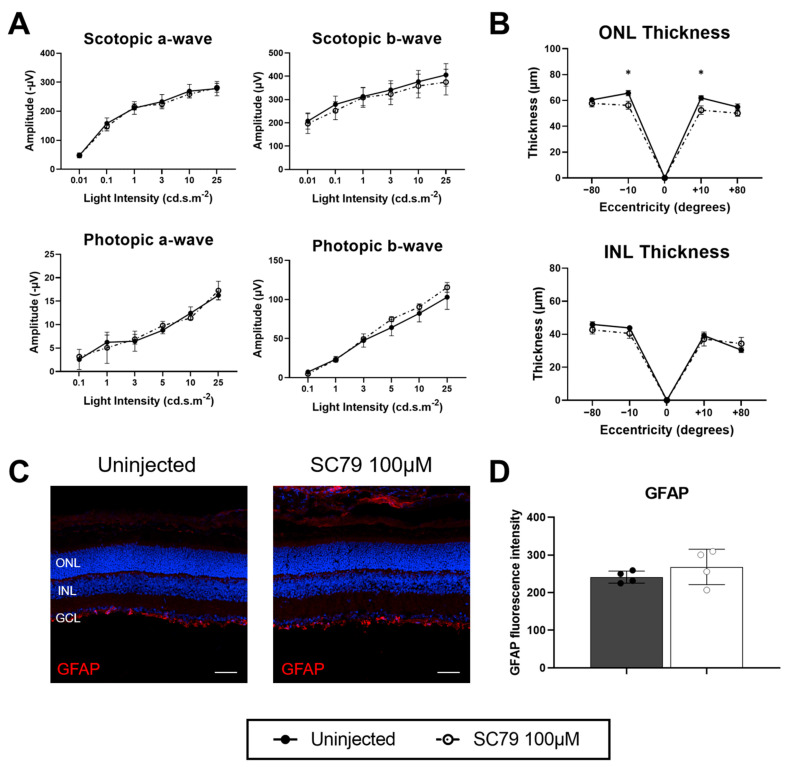
Safety testing of SC79 at a higher dose in wildtype mice. (**A**–**C**) Wildtype mice were treated at P12 with SC79 100 µM, with eyes collected for analysis at P16. (**A**) ERG responses in SC79 100 µM treated wildtype mice compared to uninjected controls. Numbers are mean ± SEM. Uninjected *n* = 4; SC79 100 µM *n* = 3. (**B**) Retinal ONL and INL thickness in uninjected and SC79 100 µM-injected wildtype mice. Numbers are mean ± SEM. Uninjected *n* = 4; SC79 100 µM *n* = 4. * *p* < 0.05. ONL: outer nuclear layer; INL: inner nuclear layer. (**C**) GFAP staining (red) showing glial reactivity in uninjected and SC79 100 µM-injected wildtype mice. Representative images were taken from the medial retina. Scale bar = 50 µm. (**D**) Analysis of relative GFAP fluorescence intensity in retinal sections from each group are shown. Fluorescence intensity was quantified by averaging three retinal sections per eye, with superior and inferior regions analysed for each section and averaged for the final value. Numbers are mean ± SEM. Uninjected *n* = 4; SC79 100 µM *n* = 4.

To investigate whether therapeutic effects of SC79 can be optimised, an increased dose of SC79 at 100 μM was administered to wildtype mice at the same age (P12) as the 10 μM dose, and results collected four days post-treatment at P16 (Figure 5). Similarly to the 10 μM dose, an increased dose of SC79 at 100 μM had no major detrimental effects on ERG responses (Figure 5A) or retinal thickness (Figure 5B) in wildtype mice. There was no observable difference in GFAP staining between uninjected and SC79-treated wildtype mice, indicating that SC79 at 100 μM did not elicit increased glial reactivity (Figure 5C), and measurement of GFAP fluorescence intensity showed groups were comparable (Figure 5D). Overall, there were minimal toxic effects of SC79 at 100 μM in wildtype mice.

### 3.4. Minimal Benefits of Increased SC79 Dose in Retinitis Pigmentosa Mice

**Figure 6:**
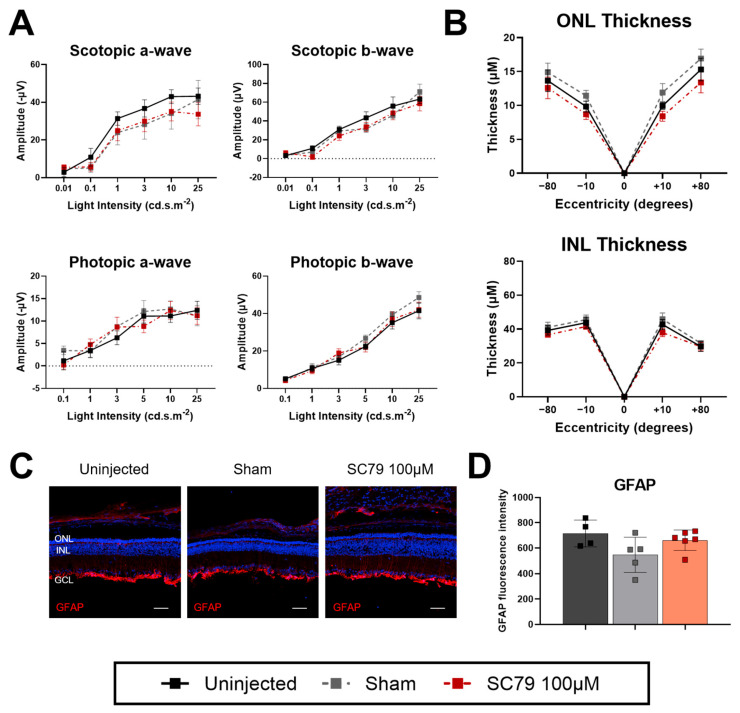
Effects of an increased dose of SC79 in *rd1*.GFP mice. (**A**) ERG responses in SC79 100 µM-treated *rd1*.GFP mice compared to uninjected controls. A black dotted line is shown at y = 0 when the lower bound of the error bars extends below zero. Uninjected *n* = 6; sham *n* = 6; SC79 100 µM *n* = 8. (**B**) Retinal ONL and INL thickness measurements in uninjected, sham-injected, and SC79 100 µM injected *rd1*.GFP mice. Numbers are mean ± SEM. Uninjected *n* = 5; sham *n* = 6; SC79 100 µM *n* = 8. ONL: outer nuclear layer; INL: inner nuclear layer. (**C**) GFAP staining (red) showing glial reactivity in uninjected, sham-injected, and SC79 100 µM injected *rd1*.GFP mice. Representative images were taken from the medial retina. Scale bar = 50 µm. (**D**) Analysis of relative GFAP fluorescence intensity in retinal sections from each group are shown. Fluorescence intensity was quantified by averaging three retinal sections per eye, with superior and inferior regions analysed for each section and averaged for the final value. Numbers are mean ± SEM. Uninjected *n* = 4; sham *n* = 5; and SC79 100 µM *n* = 6.

As an increased dosage of SC79 at 100 μM did not cause deleterious effects in wildtype mice, we tested the 100 μM dose in *rd1*.GFP mice (Figure 6). Like the lower dose, SC79 100 μM treated *rd1*.GFP mice displayed comparable scotopic ERG responses to uninjected mice (Figure 6A). There were minimal benefits to retinal thickness in *rd1*.GFP mice after treatment, though there may be a potential trend for greater retinal thickness in SC79 100 μM-treated mice in central retinal regions (Figure 6B). As treatment effect did not appear to be significantly beneficial, we did not further investigate optomotor responses. In contrast with the lower dose, the SC79-treated mice no longer had increased glial reactivity compared to uninjected *rd1*.GFP mice (Figure 6C,D).

**Figure 7:**
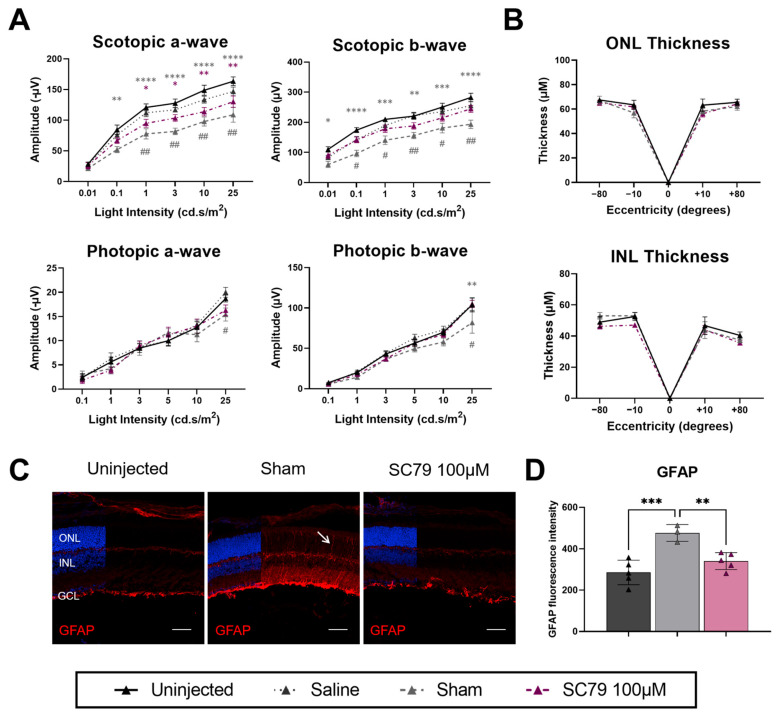
Effects of an increased dose of SC79 in *Rho*P23H.GFP mice. (**A**) ERG responses in SC79 100 µM-treated *Rho*P23H.GFP mice compared to uninjected controls. Uninjected *n* = 8; sham *n* = 5; and SC79 100 µM *n* = 6. * *p* < 0.05*,* ** *p* < 0.01, *** *p* < 0.001, and **** *p* < 0.0001. Grey asterisks indicate significance between sham and uninjected. Purple asterisks indicate significance between SC79 100 µM and uninjected. # *p* < 0.05, ## *p* < 0.01. Grey hash indicates significance between saline-injected and sham-injected groups. (**B**) Retinal ONL and INL thickness measurements in uninjected, sham-injected, and SC79 100 µM-injected *Rho*P23H.GFP. Numbers are mean ± SEM. Uninjected *n* = 6; sham *n* = 4; and SC79 100 µM *n* = 7. ONL: outer nuclear layer; INL: inner nuclear layer. (**C**) GFAP staining (red) showing glial reactivity in uninjected, sham-injected, and SC79 100 µM-injected *Rho*P23H.GFP mice. Representative images were taken from the medial retina. White arrow indicates extension of GFAP positive Müller glia processes. Scale bar = 50 µm. (**D**) Analysis of relative GFAP fluorescence intensity in retinal sections from each group are shown. Fluorescence intensity was quantified by averaging three retinal sections per eye, with superior and inferior regions analysed for each section and averaged for the final value. Numbers are mean ± SEM. Uninjected *n* = 5; sham *n* = 3; and SC79 100 µM *n* = 5. ** *p* < 0.01, *** *p* < 0.001.

The effects of increased dosage of SC79 (100 μM) were also tested in *Rho*P23H.GFP mice (Figure 7). Treatment with SC79 led to significant decreases in scotopic a-wave ERGs of *Rho*P23H.GFP mice; however, significant decreases in scotopic responses were also identified in sham-injected mice (Figure 7A). To investigate whether the DMSO within the sham was having toxic effects in *Rho*P23H.GFP, we also performed saline-only injections. It was noted that the scotopic responses of saline-injected mice were comparable to uninjected mice, though scotopic ERGs did trend lower. Interestingly, there was a significant difference in saline-injected and sham (saline containing DMSO)-injected *Rho*P23H.GFP mice, indicating that DMSO has a significant effect in reducing ERG responses within this model. Photopic responses were largely unaffected between treatment groups, except in sham-treated mice at the highest light intensity (25 cd.s/m^2^), and there were no significant changes in retinal thickness after treatment (Figure 7B). Glial reactivity at the increased SC79 dose reflected the lower dose, where sham-treated mice had an increased response, whilst SC79-treated mice had similar expression to uninjected mice (Figure 7C,D).

## 4. Discussion

Reduction in AKT activity has been implicated in various neurodegenerative diseases, including retinitis pigmentosa [5]. In the present study, we investigated the therapeutic potential of targeting AKT activation via intravitreal administration of SC79 in two distinct mouse models of retinitis pigmentosa (autosomal recessive *rd1*.GFP, and autosomal dominant *Rho*P23H.GFP). We demonstrated that SC79 treatment was well-tolerated at both 10 µM and 100 µM in wildtype mice, with no deleterious effects on retinal function or morphology. In *rd1*.GFP mice, SC79 partially rescued rod-mediated optomotor responses and moderately preserved cone photoreceptor morphology, though therapeutic benefits were not seen in *Rho*P23H.GFP mice. Our findings provide the first evidence that pharmacological activation of AKT via SC79 treatment is potentially therapeutic against photoreceptor degeneration in retinitis pigmentosa.

### 4.1. Retinal Changes After Intravitreal Sham Injections

AKT is a key regulator of neuronal survival [4], with SC79 showing therapeutic benefits in vitro in primary dopaminergic neuron cultures [8] and retinal pigment epithelial cells [9]. The current study is the first to investigate SC79 administration in IRDs. SC79 is a hydrophobic drug and requires solubilization in DMSO, which has shown to induce neuronal death within the retina at low concentrations [22]. As such, potential for drug toxicity within healthy conditions was assessed in wildtype mice alongside sham injections of the same DMSO concentration. Injection of sham led to an increase in glial reactivity, suggesting that mechanical and DMSO toxicity from the injection itself induces retinal glial activation in a healthy context. These adverse events due to mechanical trauma of intravitreal injections have also been reported previously in C57BL6/J mice [23]. In *rd1.*GFP mice, sham injections also impaired AKT protein expression and decreased activation of its downstream effector mTOR. These protein changes persisted four days after treatment, suggesting that the retina struggles to return to a baseline state in *rd1*.GFP mice, highlighting the vulnerability of the AKT pathway during retinal stress. In contrast to the sham-only injections, treatment of SC79 at 10 μM or 100 μM doses produced minimal detrimental effects in wildtype mice and restored AKT-related protein expression levels in *rd1.*GFP mice, supporting the mechanism of action of SC79 on the AKT pathway. The effects of the sham injection on the AKT pathway may have masked the neuroprotective effects of SC79, thus limiting the therapeutic potential of the treatment.

### 4.2. Model-Specific Benefits of SC79 on Rod Photoreceptors

Strikingly, even with modest therapeutic effects of SC79, where we saw an increased ONL thickness in the peripheral superior retina yet no ERG recovery, these *rd1*.GFP mice, that characteristically do not possess a rod-mediated optomotor response, were able to respond to stimuli under scotopic conditions. The robustness of the optomotor reflex may explain this discrepancy, as the reflex can be elicited from a small number of active photoreceptors, unlike full-field ERGs [24]. These findings highlight the importance of assessing ERG and optomotor motor reflex in conjunction to be able to detect treatment efficacy, particularly in cases where treatment benefits are modest. Nonetheless, targeting AKT activation for rod preservation remains a promising endeavour for autosomal recessive retinitis pigmentosa.

As for *Rho*P23H.GFP mice, previous work has shown that a reduction in photoreceptor numbers is not prominent at the early ages investigated within our study [15], though we explored whether SC79 could rescue rod-mediated ERG responses independent of rod degeneration. Interestingly, sham- and SC79-treated mice showed substantial decreases in ERG responses, but not saline-injected *Rho*P23H.GFP mice. These findings indicate that the DMSO itself, with a final concentration of 0.2% DMSO at 10 μM and 0.365% at 100 μM doses, causes a reduction in ERG responses which could in turn limit the therapeutic potential of SC79 within this mouse model. In addition, these findings may also explain why therapeutic benefits were limited in *rd1*.GFP mice, and why the higher dose did not provide additional benefits. However, why this ERG reduction occurs in *Rho*P23H.GFP mice and not in *rd1*.GFP after treatment is unclear, given the severe degeneration pattern of *rd1*.GFP mice [25]. Possibly, the already severely reduced ERG response of *rd1*.GFP mice at the investigated age makes identifying minute changes difficult to detect. Nonetheless, despite the decrease in ERG responses, there was no detrimental effect of sham or SC79 on *Rho*P23H.GFP retinal thickness, which may indicate these decreases in ERG response after treatment may be transient. Although SC79 treatment did not provide functional benefit to *Rho*P23H.GFP mice, future studies investigating later intervention timepoints that coincide with major rod degeneration may yield therapeutic benefits to photoreceptor survival and structure. This further investigation could provide insight into whether SC79 is beneficial for autosomal dominant retinitis pigmentosa. Additionally, SC79 treatment in combination with dark rearing may further enhance photoreceptor survival, which has shown therapeutic benefit in the *rd10* mouse (another autosomal recessive mouse model) [26,27] and P23H rats [28].

### 4.3. Cone Photoreceptor Morphology Preservation

Expression of AKT in cone photoreceptors has previously been shown to be important during development [29] and ageing [30], though the efficacy of targeting AKT activation in cones to prevent their degeneration in a disease context is unclear. SC79 did not improve cone-mediated ERG responses in *rd1*.GFP or *Rho*P23H.GFP mice. The effect of SC79 on cone photoreceptor numbers was only able to be evaluated in *rd1*.GFP mice, as *Rho*P23H.GFP has reportedly minimal cone death even at later adult ages [21]. Although SC79 did not improve cone numbers or function, the partial improvement of cone morphology could be indicative of the therapeutic effect of the drug. These findings in cone improvements, or the lack thereof, are mirrored in previous gene supplementation of *AKT3* in *rd10* retinitis pigmentosa mice whereby cone numbers were also unchanged after treatment [10]. In addition, this improvement in cone morphology in the superior peripheral retina aligns with potential improvements to superior peripheral retinal thickness, and could be explained as improved rod survival leading to improved cone morphology. Nonetheless, if a treatment leads to significant attenuation of rod degeneration in retinitis pigmentosa models, cones will ultimately benefit from the treatment too. Further experiments on animal models of primary cone degeneration would be of interest to evaluate the impact of SC79 exclusively on cone photoreceptors.

### 4.4. Translational Considerations

Our findings align with prior reports that targeting AKT activation can attenuate photoreceptor loss in retinitis pigmentosa [10,31]. Compared with gene therapy, pharmaceutical interventions such as SC79 offer potential advantages in cost, scalability, and ease of administration [32]. However, the limited efficacy described here emphasises the necessity to optimise treatment delivery that circumvents the need for DMSO solubilization of the drug. Systemic administration of SC79 has previously been performed and was found to be safe for treatment of liver injury in mice via intraperitoneal injections [33,34], though this route also required DMSO solubilization. Given our findings of apparent detrimental effects of DMSO on the retina, future work may explore alternative diluents for SC79. Polyethylene glycol (PEG) is a commonly used diluent for treatment in other bodily systems due to its low toxicity, though it has been shown to induce retinal degeneration when administered to the eye [35]. Alternatively, optimising the treatment modal, such as use of nanoparticle encapsulation, has recently grown within the medical research landscape due to their potential for administration of hydrophobic drugs unable to dissolve in aqueous solutions [36,37]. In addition, these nanoparticle formulations have customizable release kinetics to allow slow release of drugs over time, which could have implications for prolonging the residency of SC79 within the retina. However, effective delivery of nanoparticles to the retina have not yet been established, warranting further investigation before this treatment modal can become clinically translatable. Ensuring the mode of delivery for treatments under investigation causes as little retinal damage as possible will help isolate the true effect of SC79 and increase the reliability of the results. Therefore, future research is required to maximise therapeutic benefits before SC79 can be translated for treatment of patients with retinitis pigmentosa.

## 5. Conclusions

In conclusion, SC79 demonstrates modest efficacy in preserving photoreceptors in a recessive retinitis pigmentosa mouse model (*rd1*.GFP), but not in an autosomal dominant model (*Rho*P23H.GFP). However, the DMSO solvent presents potential imitations to treatment efficacy due to its neurotoxic properties. While these results may be promising, further investigation is needed to determine the most effective means for targeting AKT activation for neuroprotection in retinal diseases.

## Data Availability

The data collected in the current study are available from the corresponding author upon request.

## Supplementary Materials

The following supporting information can be downloaded at https://www.mdpi.com/article/10.3390/biomedicines14010195/s1, Figure S1: Representative images of cone morphology in different regions of the retina of *rd1*.GFP mice after sham or SC79 10 µM treatment.

## Abbreviations

The following abbreviations are used in this manuscript:

IRD	Inherited retinal disease
*rd1*	Retinal degeneration 1
PKB	Protein kinase B
*rd10*	Retinal degeneration 10
DMEM	Dulbecco’s Modified Eagle’s Medium
DMSO	Dimethyl sulfoxide
GFP	Green fluorescent protein
ERG	Electroretinogram
BSA	Bovine serum albumin
NGS	Normal goat serum
PBS	Phosphate-buffered saline
mTOR	Mammalian target of rapamycin
DAPI	4′,6-diamidino-2-phenylindole
SP	Superior peripheral
SC	Superior central
IC	Inferior central
IP	Inferior peripheral
ONL	Outer nuclear layer
INL	Inner nuclear layer
GCL	Ganglion cell layer
GFAP	Glial fibrillary acidic protein
H&E	Hematoxylin and eosin
