# Supplementary material for: Targeting AKT via SC79 for Photoreceptor Preservation in Retinitis Pigmentosa Mouse Models
Source: Biomedicines. 2026 Jan 15;14(1):195. doi: 10.3390/biomedicines14010195 (PMC12839193; doi:10.3390/biomedicines14010195)
Supplement: Supplementary file 1 [file biomedicines-14-00195-s001.zip › biomedicines-3943723-supplementary.pdf]

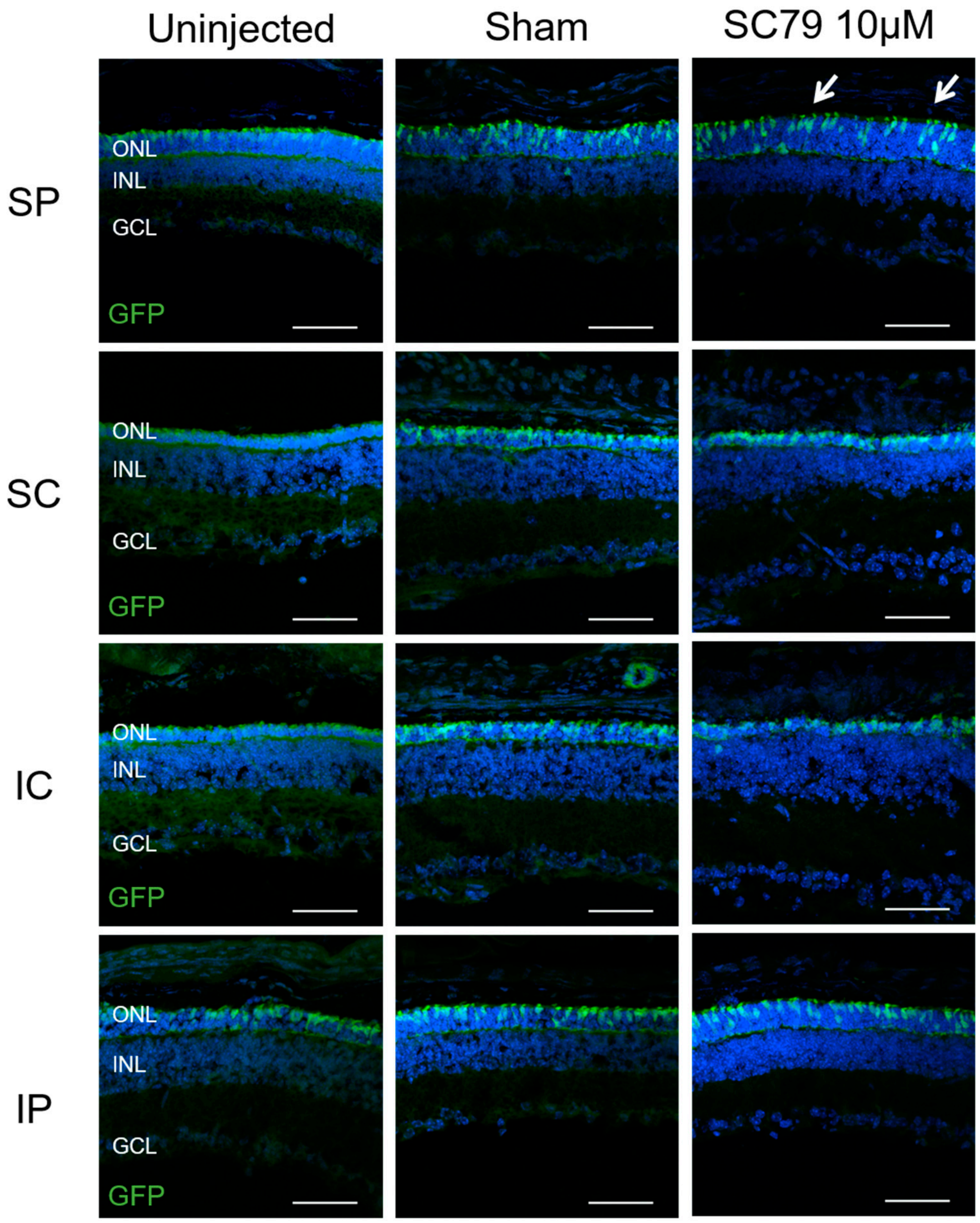


**Supplementary Figure S1.** Representative images of cone morphology in different regions of the retina of *rd1*.GFP mice after sham or SC79 10 µM treatment. White arrows indicate partial preservation of cone inner segments. Scale bar = 50 µm.
